# Bilateral Mooren's Ulcer With Corneal Perforation in Two Women of Different Ethnicities: A Report of Two Cases

**DOI:** 10.7759/cureus.112503

**Published:** 2026-07-12

**Authors:** Alessandra Balestrazzi, Paolo Michieletto, Marzia Michieletto

**Affiliations:** 1 Ophthalmology, Azienda Sanitaria Locale (ASL) Roma 2, Rome, ITA; 2 Ophthalmology, Azienda Sanitaria Locale (ASL) Roma 1, Rome, ITA; 3 Ophthalmology, Agostino Gemelli University Polyclinic, Rome, ITA

**Keywords:** case report, conjunctival resection, corneal perforation, keratoplasty, mooren’s ulcer, systemic immunosuppression

## Abstract

We present two patients with bilateral Mooren’s ulcer complicated by corneal perforation and describe their surgical and systemic management.

A 55-year-old Caucasian woman (Case 1) and a 55-year-old African woman (Case 2) presented with bilateral Mooren’s ulcer. Both had corneal perforation in the right eye and impending perforation in the left eye. Visual acuity in the right eye was reduced due to nasal pupillary displacement and posterior capsular opacification in Case 1 and cataract in Case 2. Both patients had previously undergone a corneal-conjunctival patch procedure in the right eye, but the diagnosis of Mooren’s ulcer had not been established prior to referral.

Surgical management included lamellar keratoplasty in Case 1 and penetrating keratoplasty with selective conjunctival resection in Case 2. Systemic immunosuppression with mycophenolate mofetil (MMF) was administered in both cases.

In Case 1, posterior capsulotomy improved visual acuity to 20/50 with spectacles and to 20/30 with scleral contact lenses. At follow-up, Case 1 showed stability in both eyes, 2 years after lamellar keratoplasty in the right eye and 20 months after conjunctival resection in the left eye. Case 2 had a shorter follow-up, with stability at six months in the right eye and at three months in the left eye.

These cases highlight the aggressive bilateral nature of Mooren’s ulcer, particularly in the African population, and underscore the importance of individualized surgical and systemic management. Early recognition and combined therapy, including keratoplasty, conjunctival resection, and systemic immunosuppression, can stabilize the disease and optimize visual outcomes despite the initial severe presentation.

## Introduction

According to some studies [1,2], Mooren’s ulcer is a rare, idiopathic form of peripheral ulcerative keratitis characterized by progressive stromal thinning, severe hyperemia, intense ocular pain, and potential corneal perforation. It is considered a localized autoimmune disorder in the absence of systemic inflammatory disease. The pathogenesis of Mooren’s ulcer remains incompletely understood, and its diagnosis continues to rely largely on exclusion.

Although the underlying mechanisms remain elusive, Mooren’s ulcer is generally recognized as an autoimmune disease involving both cellular and humoral immune pathways. The cornea and adjacent conjunctiva near ulcer sites have been shown to contain corneal-associated antigens (CO-Ag), human leukocyte antigen (HLA) class II molecules, and infiltrating inflammatory cells [1].

Histopathologic studies have demonstrated marked infiltration of T lymphocytes, along with B cells, macrophages, neutrophils, natural killer cells, and mast cells. Elevated circulating immune complexes may further enhance inflammatory cell recruitment and collagenase release, resulting in stromal lysis and progressive ulceration [1].

Clinical severity varies widely, ranging from indolent unilateral disease to aggressive bilateral involvement, with more severe presentations often reported in patients of African and Asian ancestry [1,2]. The incidence, clinical characteristics, and disease severity also vary significantly across geographical regions and among different racial groups.

Management is challenging and often requires a combination of surgical intervention and systemic immunosuppressive therapy [3-7].

This report describes two patients with similar clinical presentations but different ethnic backgrounds and their management outcomes. In both cases, the patients had no significant systemic disease. Both had previously been treated at other institutions; however, Mooren’s ulcer had not been diagnosed despite the presence of nasal corneal perforation in the right eye, severe intractable pain, photophobia, and early involvement of the nasal sector of the fellow eye. Both patients underwent extensive immunological and rheumatological evaluations, which revealed no evidence of systemic disease. Based on the clinical presentation and after excluding other potential causes, we diagnosed Mooren’s ulcer.

## Case presentation

Case 1

**Figure 1:**
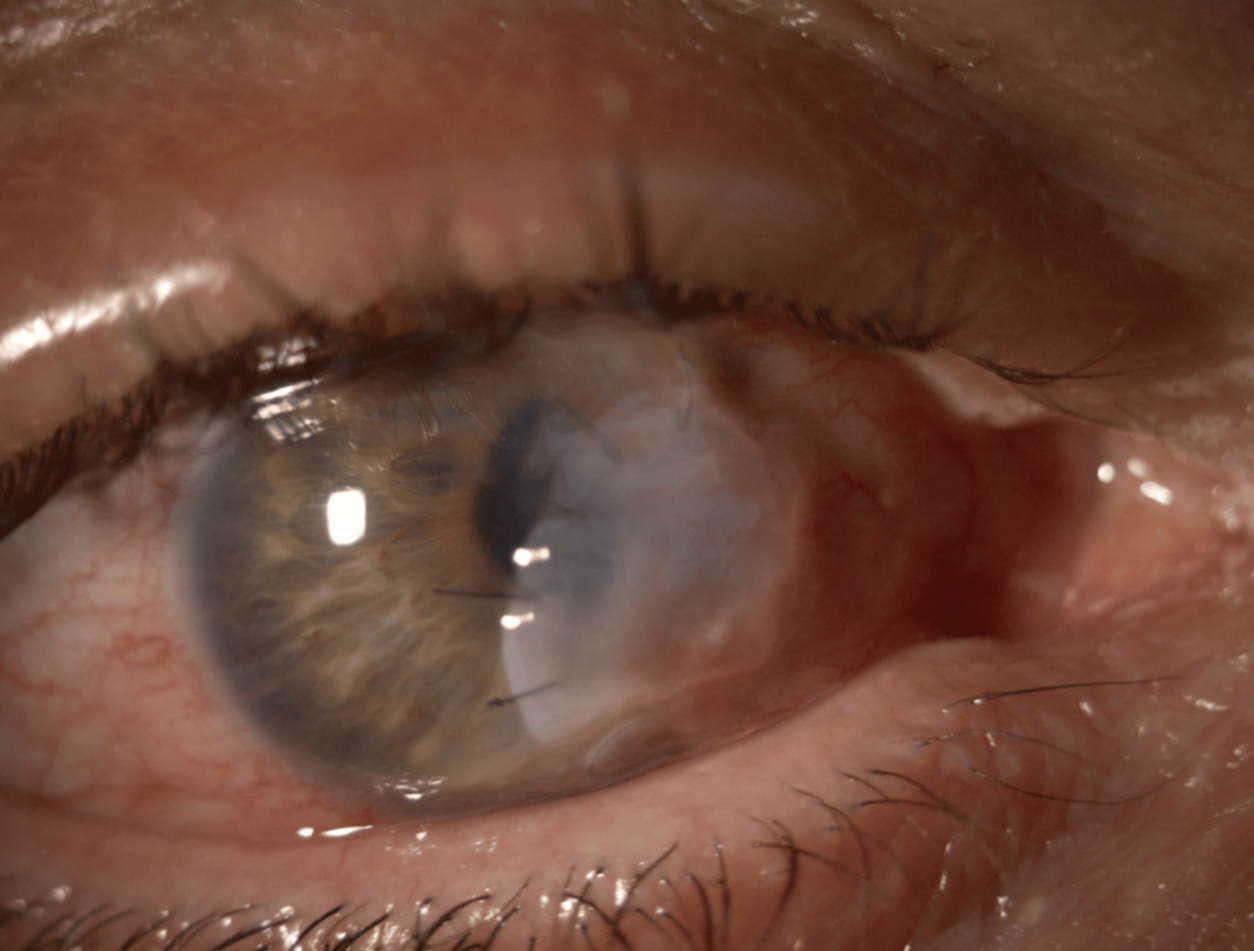
Right eye at presentation, showing a previous corneal-conjunctival patch procedure.

A 55-year-old Caucasian woman presented with severe ocular pain, photophobia, and progressive visual impairment. Slit-lamp examination revealed evidence of a previous corneal-conjunctival patch procedure performed for a nasal corneal perforation in the right eye (Figure 1).

**Figure 2:**
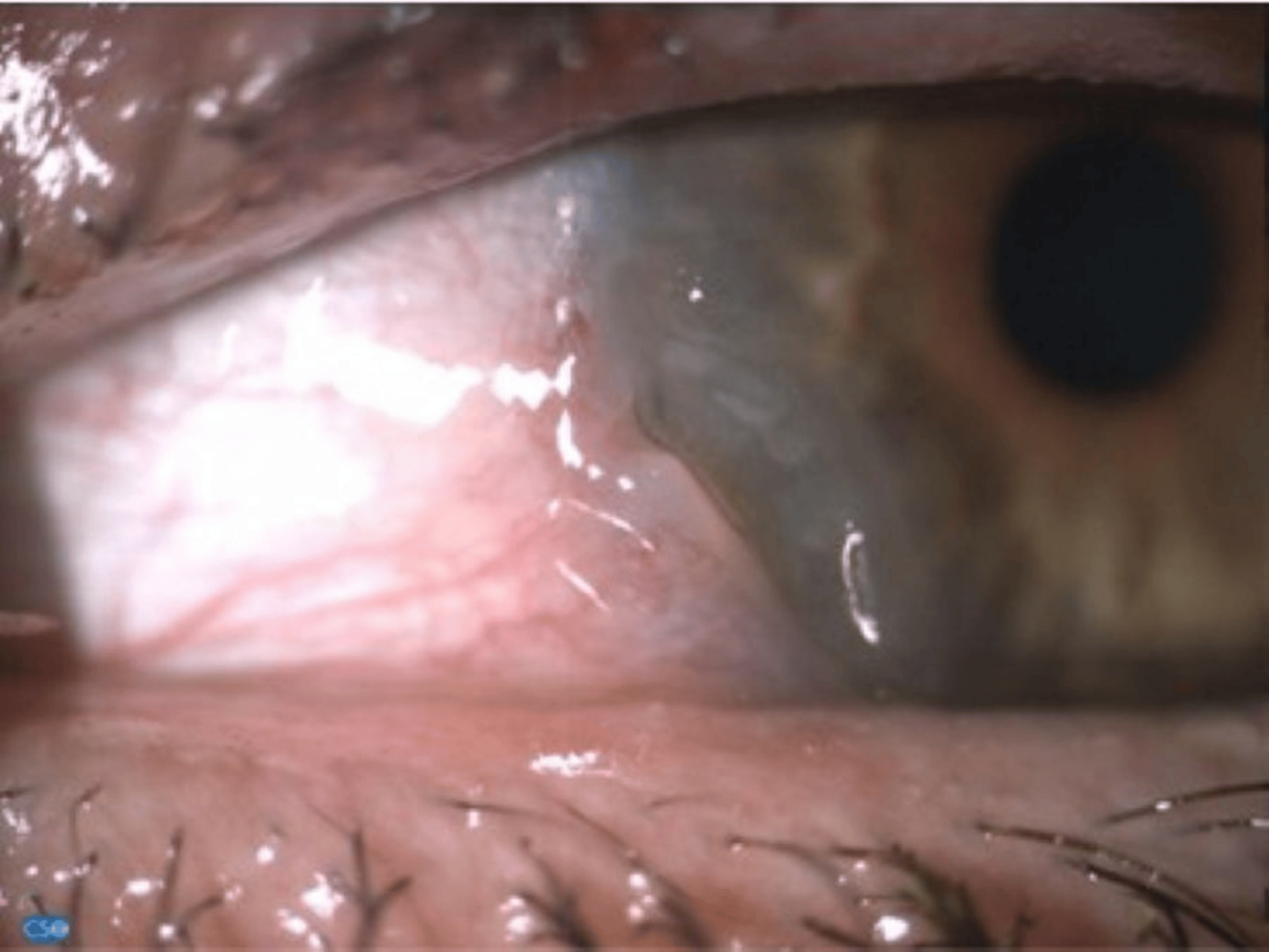
Left eye before conjunctival resection, showing impending corneal perforation.

**Figure 3:**
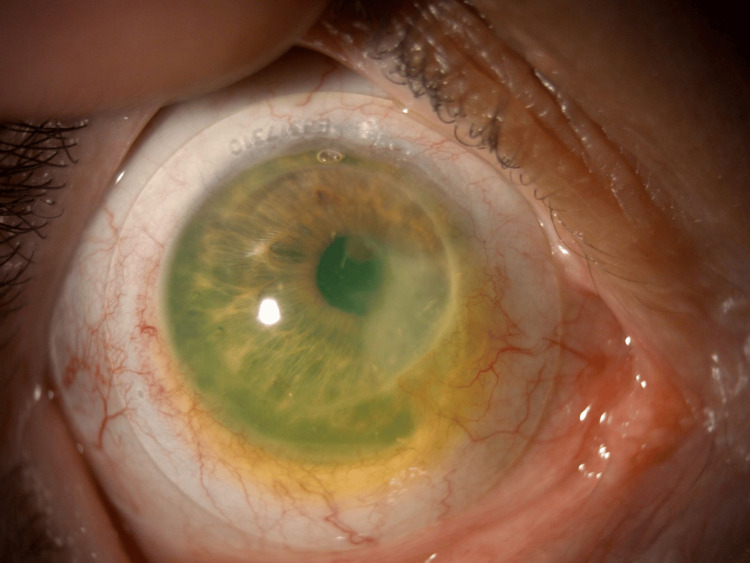
Right eye at the two-year follow-up after lamellar keratoplasty, showing a stable corneal surface with a scleral contact lens.

**Figure 4:**
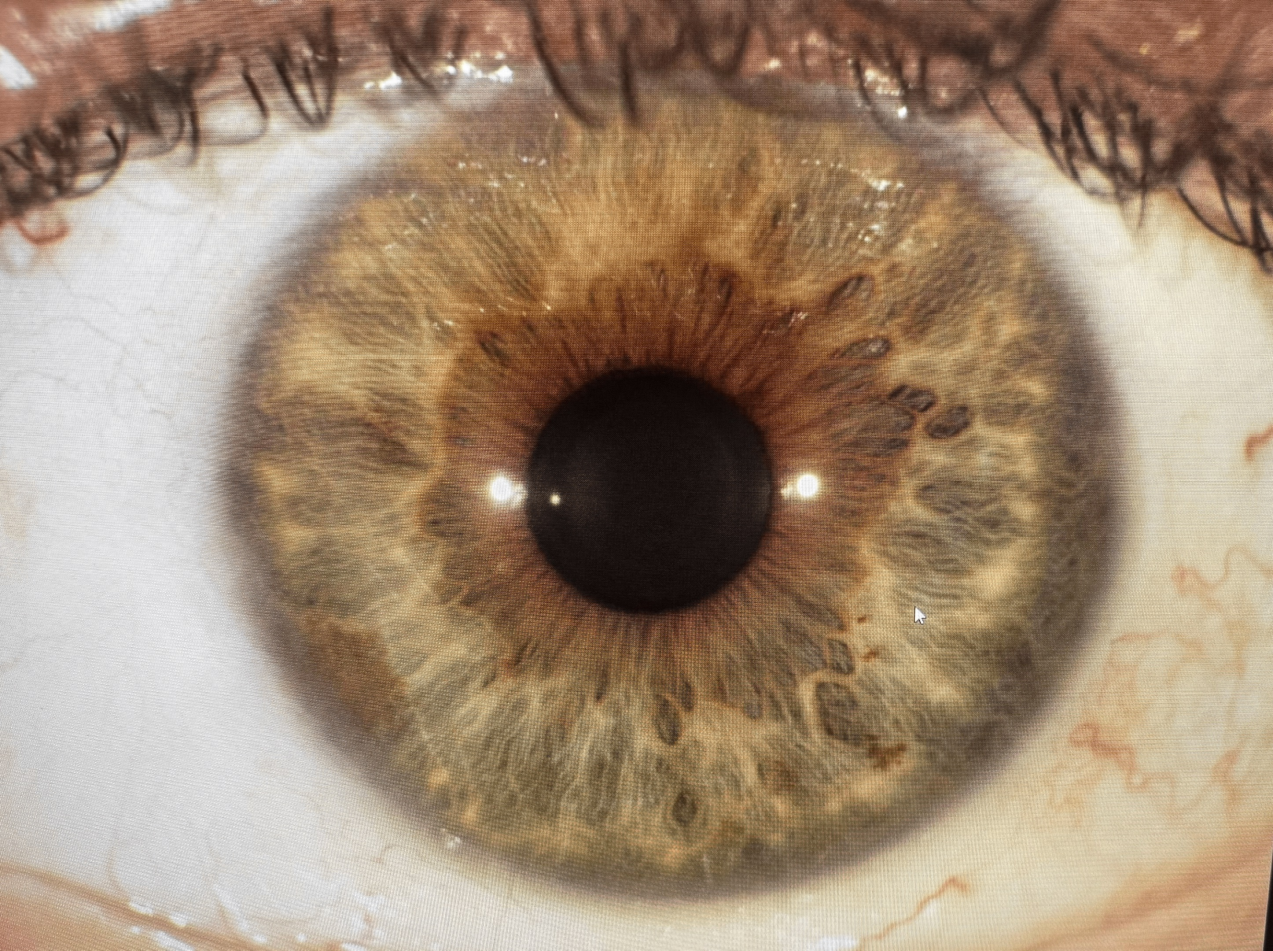
Left eye at the 20-month follow-up after conjunctival resection, showing a stable corneal surface.

Lamellar keratoplasty was performed in the right eye. Two days after surgery, initial corneal melting was observed. Oral prednisone (50 mg/day) was initiated, and a therapeutic contact lens was applied. Topical treatment with betamethasone eye drops, antibiotic eye drops, lubricating eye drops, and re-epithelializing eye drops was also started. Because the response to this treatment was limited, oral mycophenolate was initiated. Mycophenolate was selected because it selectively targets lymphocytes and is effective and easy to administer. The addition of mycophenolate improved the corneal melting and stabilized the clinical condition. The patient received mycophenolate at a dose of 500 mg/day for one year. Due to progression of peripheral ulceration in the left eye (Figure 2), nasal and inferonasal conjunctival resection was subsequently performed in the areas of hyperemia and corneal thinning. Following the procedure, the patient reported marked improvement, with a significant reduction in hyperemia, photophobia, and tearing in both eyes. At follow-up, both eyes remained stable: 2 years after lamellar keratoplasty in the right eye and 20 months after conjunctival resection in the left eye (Figures 3, 4).

Best-corrected visual acuity (BCVA) in the right eye was 20/50 with spectacles and improved to 20/30 with a contact lens. The reduced visual acuity was attributed to nasal pupillary displacement and posterior capsular opacification, which was treated with Nd laser posterior capsulotomy. BCVA in the left eye was 20/20.

Case 2

**Figure 5:**
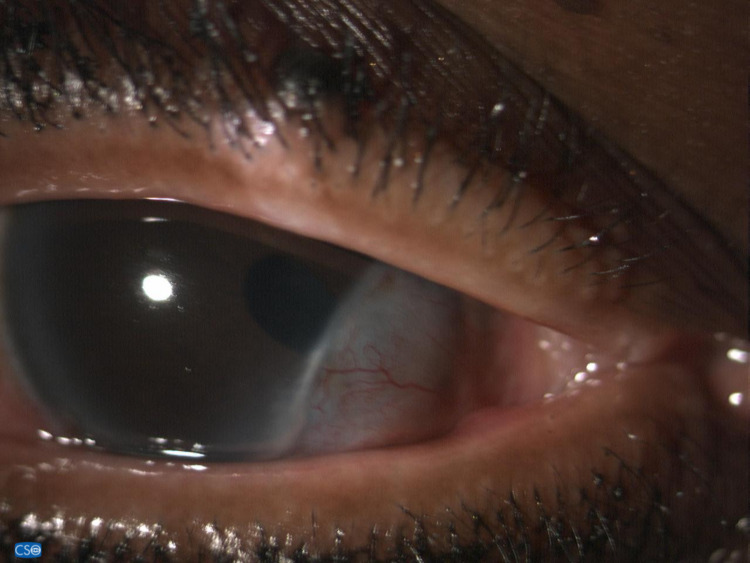
Right eye at presentation, showing a previous corneal-conjunctival patch procedure for a nasal corneal perforation.

A 55-year-old African woman presented with rapidly progressive bilateral peripheral ulcerative keratitis. The right eye showed evidence of a previous corneal-conjunctival patch procedure performed for a nasal corneal perforation (Figure 5).

**Figure 6:**
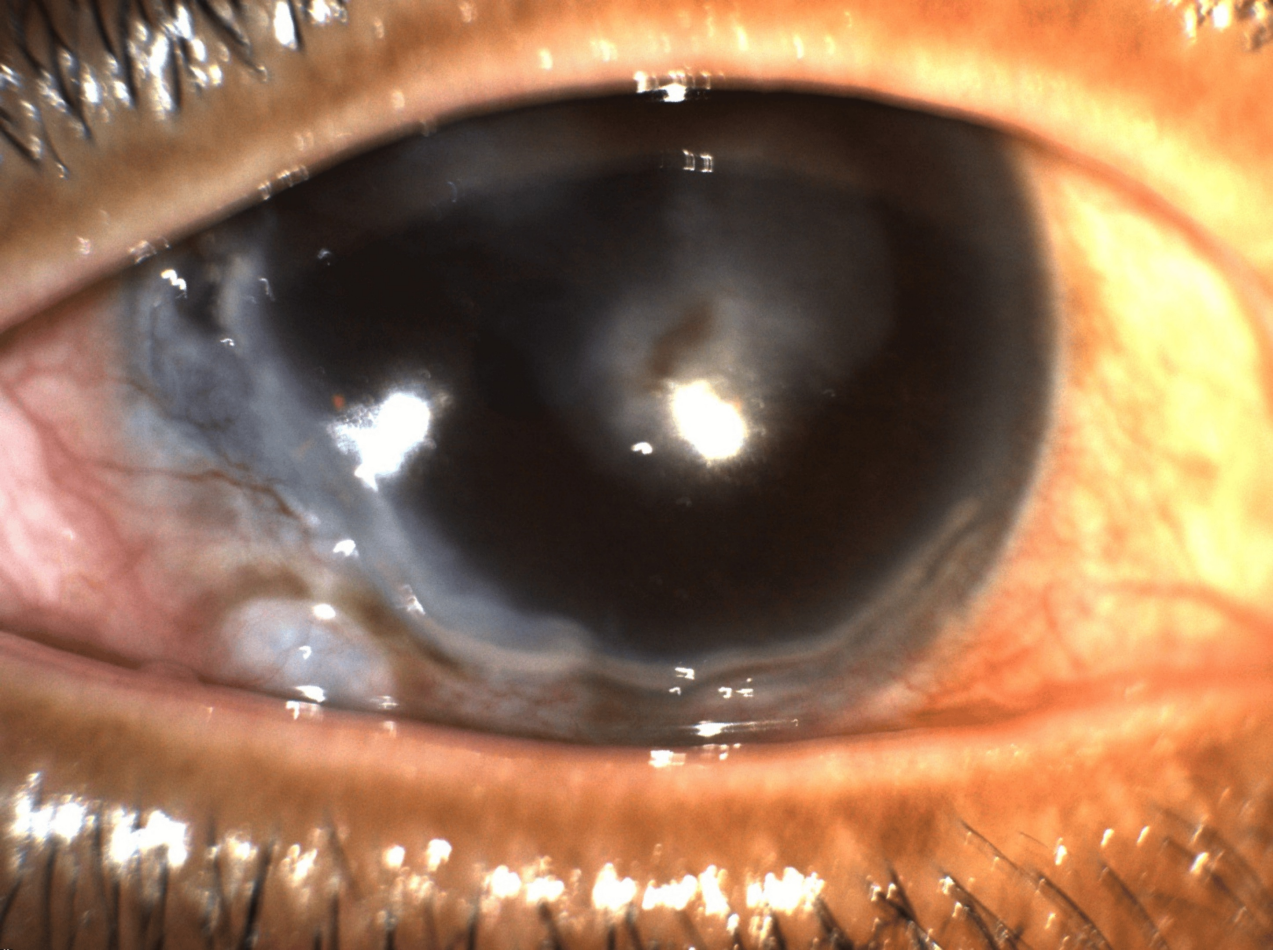
Left eye showing impending corneal perforation.

The left eye showed impending corneal perforation (Figure 6).

BCVA in the right eye was reduced due to nasal pupillary displacement and cataract. The patient had no systemic disease, was not taking any medications, and blood test results, including investigations for autoimmune diseases, were negative. Therefore, other causes of peripheral ulcerative keratitis (PUK) were excluded. Based on the clinical similarity to Case 1, we diagnosed Mooren’s ulcer and initiated treatment with corticosteroids, topical re-epithelializing agents, and systemic mycophenolate, resulting in partial clinical improvement.

**Figure 7:**
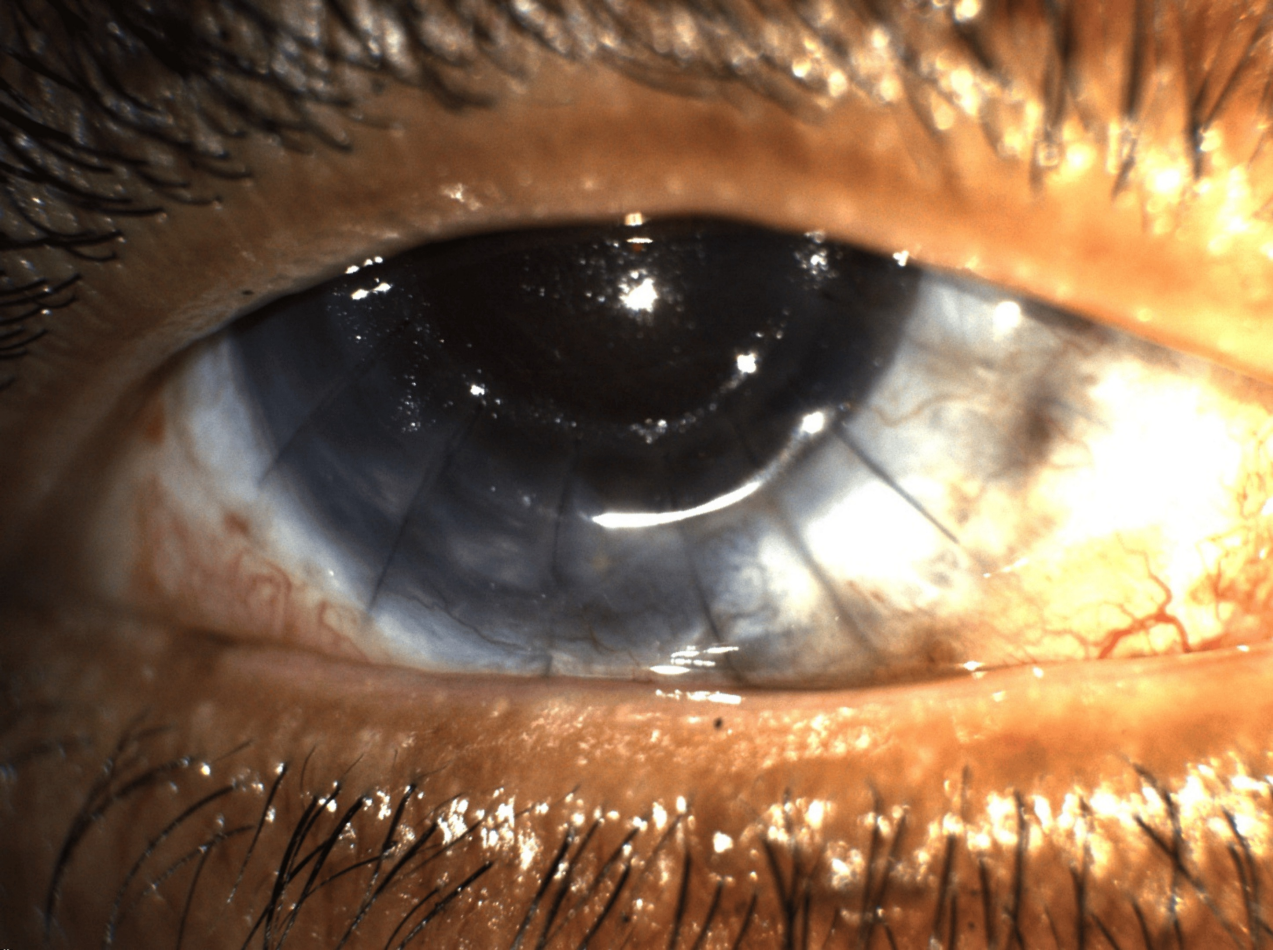
Right eye after penetrating keratoplasty, showing a stable corneal surface.

**Figure 8:**
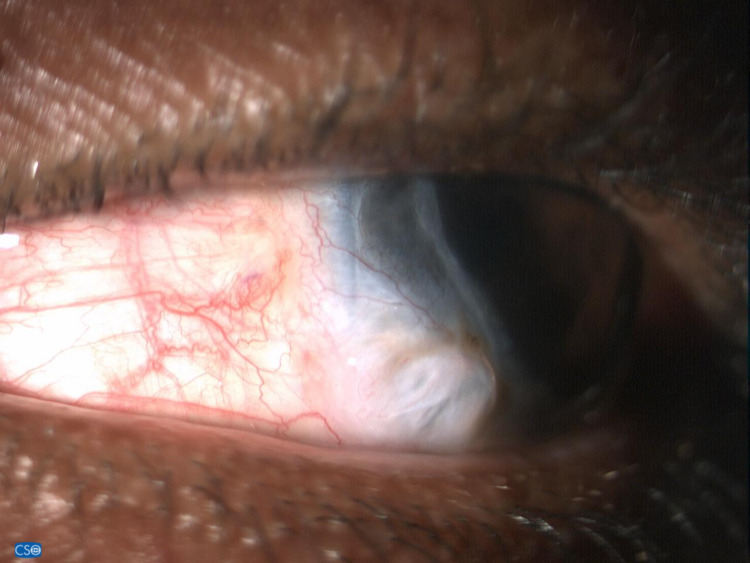
Left eye after conjunctival resection, showing a stable corneal surface.

Surgical management included penetrating keratoplasty in the right eye combined with nasal and inferior conjunctival resection, as these were the areas with the most marked hyperemia and conjunctival swelling. No conjunctival resection was initially performed in the left eye. During follow-up, the patient developed a microbial corneal ulcer in the left eye caused by *Pseudomonas aeruginosa*, associated with the use of a therapeutic contact lens that was replaced monthly. The infection was successfully treated with targeted topical antimicrobial therapy and close clinical monitoring. After resolution of the microbial ulcer, nasal and inferior conjunctival resection was performed in the left eye because of persistent severe photophobia and paralimbal corneal thinning in the affected areas. Follow-up demonstrated stable findings at three and six months in the right eye and at one and three months in the left eye (Figures 7, 8).

BCVA in the right eye was reduced due to high astigmatism, nasal pupillary displacement, and cataract. Removal of the corneal sutures, cataract surgery, and possible surgical pupillary enlargement are planned to optimize visual rehabilitation.

BCVA in the left eye was reduced due to a paracentral corneal scar but improved after nasal and inferior conjunctival resection to 20/40 without correction and 20/25 with correction.

Both patients were treated with systemic immunosuppressive therapy using MMF at a dose of 250 mg twice daily. In Case 1, treatment was discontinued after one year because the patient reported paresthesia and altered sensation in the lower limbs during therapy. In Case 2, the treatment was well-tolerated. During follow-up, both patients demonstrated reduced inflammatory activity, improvement in conjunctival redness and eyelid opening, stabilization of corneal thinning, and no further episodes of corneal perforation.

## Discussion

These cases illustrate the severe bilateral nature of Mooren’s ulcer and emphasize the importance of individualized surgical strategies. Corneal perforation required lamellar or penetrating keratoplasty, while selective conjunctival resection was tailored according to disease activity and laterality to suppress local immune-mediated inflammation. The marked symptomatic improvement observed after conjunctival resection is consistent with previous reports [3-6] supporting this procedure in Mooren’s ulcer.

Mooren’s ulcer is sustained by a local autoimmune response, and conjunctival resection interrupts this inflammatory process, thereby reducing the immune-mediated attack on the cornea.

The goals of conjunctival resection are to remove immunologically active conjunctival tissue, reduce the local release of proteolytic enzymes, slow the circumferential and centripetal progression of the ulcer, and enhance the effectiveness of medical immunosuppressive therapy.

Mycophenolate selectively inhibits the proliferation of T and B lymphocytes, reduces the production of autoantibodies, has lower long-term toxicity than many other immunosuppressive agents, helps control disease progression, reduces relapses, and facilitates steroid tapering or discontinuation.

The favorable response to systemic MMF supports its effectiveness in controlling Mooren’s ulcer and other ocular surface inflammatory disorders. MMF has been reported to be an effective and relatively safe steroid-sparing immunosuppressive agent when topical therapy alone is insufficient [7]. Initial systemic corticosteroids are commonly used to achieve rapid suppression of inflammation, followed by steroid-sparing agents such as methotrexate or MMF for long-term disease control [7].

The occurrence of a contact lens-associated *P. aeruginosa* infection in the left eye of Case 2 highlights the increased risk of infectious complications in eyes with compromised corneal integrity, particularly in the setting of therapeutic contact lens wear and systemic immunosuppression. After resolution of the infection, conjunctival resection was performed to further control local immunologic activity. This procedure is believed to reduce the exposure of corneal antigens to the immune system, thereby decreasing inflammatory cell infiltration and promoting corneal healing.

The rapid progression observed after previous corneal-conjunctival patch procedures in both patients is noteworthy, as worsening of Mooren’s ulcer following conjunctival flap surgery has been reported.

Follow-up demonstrated long-term stability in Case 1 (2 years in the right eye and 20 months in the left eye) and shorter-term stability in Case 2 (6 months in the right eye and 3 months in the left eye), supporting the effectiveness of the combined surgical and medical approach.

## Conclusions

Mooren’s ulcer may present with aggressive, asymmetrical bilateral involvement and corneal perforation. A customized combination of keratoplasty, selective conjunctival resection, and systemic immunosuppression can achieve disease stabilization and improve visual outcomes. A notable feature of these cases was that the diagnosis of Mooren’s ulcer was not established until both eyes were involved. In both patients, the condition was recognized only after the fellow eye developed signs of impending corneal perforation in the nasal sector. Although systemic therapy with MMF led to an overall improvement in the clinical course, a more rapid and marked resolution of severe photophobia and ocular pain was achieved only after conjunctival resection was performed in the sectors corresponding to corneal thinning and severe hyperemia. This finding underscores the critical role of conjunctival inflammation in both symptom severity and local disease activity.

## References

[1] Ou S, Zhang Y, Feng Y (2025). Mooren's ulcer: a multifactorial autoimmune peripheral ulcerative keratitis and current treatment protocols. Front Med (Lausanne).

[2] Zelefsky JR, Srinivasan M, Cunningham ET Jr (2011). Mooren’s ulcer. Expert Rev Ophthalmol.

[3] Ashar JN, Mathur A, Sangwan VS (2013). Immunosuppression for Mooren's ulcer: evaluation of the stepladder approach--topical, oral and intravenous immunosuppressive agents. Br J Ophthalmol.

[4] Kim DH, Kim MK, Wee WR, Oh JY (2016). Mooren's ulcer in a cornea referral practice in Korea. Ocul Immunol Inflamm.

[5] Al-Noufali M, Al-Habsi S, Imtiaz M, Al-Julandani N, Al-Yarabi M (2026). Conjunctival resection for Mooren's ulcer refractory to medical therapy: a case report. Ophthalmology.

[6] Furundaoturan O, Akçay P, Selver OB (2022). Use of systemic mycophenolate mofetil therapy in ocular surface inflammatory pathologies at the initiative and responsibility of the ophthalmologist. Middle East Afr J Ophthalmol.

[7] Maleki A, Valerio T, Massoudi Y, Ruggeri ML, Foster CS, Anesi SD (2024). Updates on systemic immunomodulation in peripheral ulcerative keratitis. Vision (Basel).

